# Investigating associations between the built environment and physical activity among older people in 20 UK towns

**DOI:** 10.1136/jech-2017-209440

**Published:** 2017-11-24

**Authors:** Sophie Hawkesworth, Richard J Silverwood, Ben Armstrong, Triantafyllos Pliakas, Kiran Nanchalal, Barbara J Jefferis, Claudio Sartini, Antoinette A Amuzu, S Goya Wannamethee, Sheena E Ramsay, Juan-Pablo Casas, Richard W Morris, Peter H Whincup, Karen Lock

**Affiliations:** 1 Faculty of Public Health and Policy, London School of Hygiene and Tropical Medicine, London, UK; 2 Faculty of Epidemiology and Population Health, London School of Hygiene and Tropical Medicine, London, UK; 3 UCL Department of Primary Care & Population Health, UCL Medical School, London, UK; 4 UCL Physical Activity Research Group, London, UK; 5 Farr Institute of Health Informatics, Faculty of Population Health Sciences, London, UK; 6 School of Social and Community Medicine, University of Bristol, Bristol, UK; 7 Population Health Research Institute, St George’s, University of London, London, UK

**Keywords:** physical activity, neighborhood/place, ageing

## Abstract

**Background:**

Policy initiatives such as WHO Age Friendly Cities recognise the importance of the urban environment for improving health of older people, who have both low physical activity (PA) levels and greater dependence on local neighbourhoods. Previous research in this age group is limited and rarely uses objective measures of either PA or the environment.

**Methods:**

We investigated the association between objectively measured PA (Actigraph GT3x accelerometers) and multiple dimensions of the built environment, using a cross-sectional multilevel linear regression analysis. Exposures were captured by a novel foot-based audit tool that recorded fine-detail neighbourhood features relevant to PA in older adults, and routine data.

**Results:**

795 men and 638 women aged 69–92 years from two national cohorts, covering 20 British towns, were included in the analysis. Median time in moderate to vigorous PA (MVPA) was 27.9 (lower quartile: 13.8, upper quartile: 50.4) minutes per day. There was little evidence of associations between any of the physical environmental domains (eg, road and path quality defined by latent class analysis; number of bus stops; area aesthetics; density of shops and services; amount of green space) and MVPA. However, analysis of area-level income deprivation suggests that the social environment may be associated with PA in this age group.

**Conclusions:**

Although small effect sizes cannot be discounted, this study suggests that older individuals are less affected by their local physical environment and more by social environmental factors, reflecting both the functional heterogeneity of this age group and the varying nature of their activity spaces.

## Introduction

Physical inactivity is an important risk factor for a range of non-communicable diseases across the life course.^1^ Physical activity (PA) is particularly important for older people’s physical and psychological health.^2–5^ However, older people have low PA and rarely achieve recommended levels.^6^ Only 15% of men and 10% of women meet moderate to vigorous PA guidelines in the UK.^7^ There is increasing policy interest in the role of the physical environment in determining PA, although to date there is less evidence from older age groups.^8–10^

Socioecological frameworks propose that PA is influenced by complex interactions between individual, social and environmental factors.^11^ Older people may be particularly sensitive to neighbourhood influences that can be magnified by deteriorating physical and cognitive functioning,^12 13^ and because they spend more time locally.^14^ A variety of contextual environmental features are considered important.^15–17^ While earlier reviews had been inconclusive,^9 10^ the most recent systematic review of the built environment and PA in older adults found strong evidence of positive associations between neighbourhood walkability, access to destinations and services, personal safety from crime and PA, although associations varied by measurement type.^18^ However, these reviews also identified some important limitations of the evidence base. Many studies of the built environment have focused on discrete environmental exposures, such as walkability or green spaces, or environmental perception, though many environmental factors may influence PA. Research questions are often driven by what can be estimated from routine data rather than relevance to public health. Qualitative studies highlight the importance of more nuanced research approaches to understand environment–activity associations.^19^

This research aimed to explore a wide range of complex environmental attributes that hypothetically influence older adults’ PA in their neighbourhood environments. The study objective was to investigate the association between objectively measured PA and the neighbourhood environment captured using fine-detail, systematic and objective environmental measurement incorporating audit tool assessments developed specifically for older people in 20 UK towns.

## Methods

This cross-sectional study was nested within two national cohorts that defined the built environment areas assessed. The British Regional Heart Study (BRHS) was established in 1978–1980 recruiting 7735 men from primary care in 24 British towns into an ongoing prospective nationally representative cohort study.^20^ In 1999–2000, a parallel women’s cohort, the British Womens’ Heart and Health Study (BWHHS), was established recruiting a total of 4286 women in 23 towns.^21^ In 2010–2012, 6529 survivors from both cohorts in all towns (3292 men and 3237 women) were eligible to participate in a study of objectively measured PA. Ethical approval was provided by the National Research Ethics Service Committee for London.

Details of the PA measurement have been published elsewhere.^7^ Briefly, participants were asked to wear a GT3x accelerometer (Actigraph, Pensacola, Florida) over the right hip for 7 days during waking hours. Data were processed using standard procedures.^7^ Valid wear days were defined as ≥600 min wear time; participants with three or more valid days were included in the analysis.^7^ Minutes per day spent in PA of various intensity was categorised using count-per-minute threshold values developed for older adults: 100–1040 for light-intensity PA (LIPA; 1.5–3 metabolic equivalent of task (MET)) and >1040 for MVPA (≥3 MET).^22^

Potential confounding factors and effect modifiers were available from previous surveys: socioeconomic status was defined at cohort baseline as the longest held occupation (men) or the highest occupational class of the study member and their husband (women); age was defined by date of birth, and long-standing illness, disability or infirmity was self-reported.

### Environmental data

A neighbourhood environment audit tool (Older People’s Environments and CVD Risk (OPECR) tool) was developed to capture neighbourhood features relevant to CVD risk factor behaviours in older adults, described in detail elsewhere.^23^ The tool consists of 100 indicators including built environment features relevant to PA such as street connectivity, traffic volume and pavement quality. Foot-based audits were conducted in 20 BWHHS and 19 BRHS study towns across the UK. Lower Layer Super Output Areas (LSOA) (geographical areas containing between 1000 and 3000 individuals) were chosen as the unit of data collection in England,^24^ while the equivalent datazones (DZ) were used in Scotland (areas containing 500–1000 individuals).^25^

The audit tool was developed in 2009 and piloted in two study towns, with the remaining towns audited between 2012 and 2014.^23^ Trained fieldworkers worked across multiple study towns to maximise data collection consistency. Fieldworkers worked in pairs systematically recording all relevant aspects of the OPECR tool for both sides of a road or ‘segment’. Interobserver reliability was found to be high with agreement ranging from ‘substantial’ for more subjective variables to ‘excellent’ for objective estimates such as traffic counts.^23^ All roads within an LSOA/DZ were audited and considered one data collection ‘segment’. Any urban LSOA/DZ where at least one cohort member lived were eligible for inclusion in the audit but large semirural areas were excluded if they were not contiguous with the study town or if they included ≤3 cohort members.

The current analysis conducted in 2015 used audit data on a number of environmental domains captured by the tool: road and pavement/sidewalk quality, bus stops, neighbourhood aesthetics, shops, services and greenspace. Variables collected as ordered categorical data (road quality) were aggregated at area level by assigning each category a score from 0 to n (for an n+1 level categorical variable) and calculating the mean across the LSOA/DZ. Variables collected as count data (road crossings, bus stops, shops) were summed across, and standardised by, LSOA/DZ. Variables collected as binary data (presence/absence of greenspace) were averaged across the LSOA/DZ to give a proportion.

Several routine data sources were also used in the analysis. Mid-year population estimates for 2010 were obtained from the Office of National Statistics^5^ and the Scottish Neighbourhood Statistics^26^; estimates were used to generate area-level population density per km^2^ smoothed using a 5 km radius buffer. Area social deprivation was defined from the income deprivation domain of the 2010 Index of Multiple Deprivation (IMD)^27^ and the 2009 Scottish Index of Multiple Deprivation (SIMD).^28^ Area crime levels were also extracted from the IMD and SIMD; for both these indices, LSOA/DZ rank was used to define the relative deprivation. Street connectivity was used as a standard walkability index; data on the number of road nodes/interconnections within an LSOA/DZ were obtained from the 2015 Ordinance Survey (Digimap Meridian 2 National) and used to generate street connectivity defined as number of intersections per km^2^.

### Statistical analysis

Study members were eligible to be included in the analysis if their LSOA/DZ of residence was covered by the foot-based environmental audit and they did not self-report having ‘severe’ or worse difficulties getting about outdoors.

To reduce the dimensionality of the data relating to road and path quality, a latent class analysis (LCA) was conducted (details in online supplementary material, tables S1-S3). Ten variables related to road and path quality, collected by the foot-based audit, were included in the LCA: ‘quality of pavement’, ‘lowered curbs’, ‘barriers on pavement’, ‘pavement width’, ‘pedestrian traffic’, ‘road use’, ‘road connectivity’, ‘traffic calming measures’, ‘lamp posts’ and ‘road crossings’. A three-class model was considered most appropriate with classes characterised as ‘poor quality walking environment’ (9.9% of segments), ‘medium quality walking environment’ (57.0%) and ‘good quality walking environment’ (33.1%). The three classes were assigned a score (0, 1 and 2), the mean of which across the LSOA/DZ is referred to as the ‘road quality score’.

10.1136/jech-2017-209440.supp1 Supplementary file 1

Time in MVPA (min/day) was considered the primary outcome; as the distribution was heavily right skewed, it was analysed on the log scale then transformed back to give an estimate of the relative difference. Secondary outcomes were time in LIPA (min/day analysed on the original scale) and total step count per day (analysed on the log scale). Multilevel linear regression models were used; the three-level structure underlying the data (study members nested within LSOAs/DZ nested within towns) was acknowledged, with random intercepts at the LSOA/DZ and town levels.

For each outcome and area-level exposure of interest (road quality score, transport, aesthetics, shops and services, green areas, income, crime, walkability, population density), sequentially adjusted models were fitted as follows: (i) minimally adjusted (season of accelerometer measurement, average monitor wear time); (ii) additionally adjusted for confounders (sex, age, adult social class, long-standing illness, disability or infirmity, country); (iii) additionally adjusted for all other area-level exposures of interest. Interactions between each exposure of interest and sex and season of accelerometer measurement were examined using Wald tests in the minimally adjusted model. We also examined effect modification by car usage. As a sensitivity analysis, we analysed the individual components of the road quality score with all primary and secondary outcomes using an analogous approach. All analyses were conducted using Stata V.14.0 (Stata).

## Results

**Figure 1:**
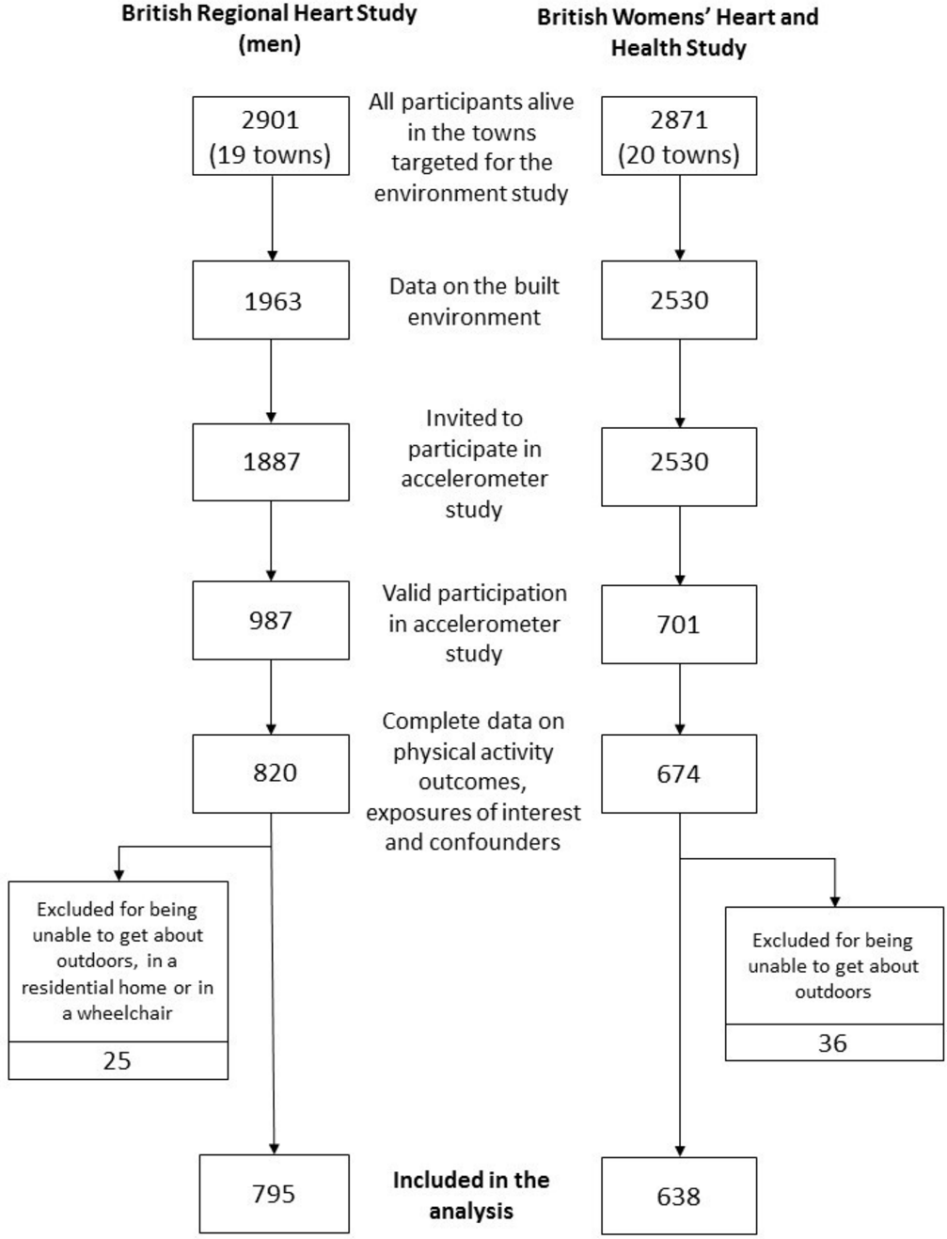
Flow of participants from the two cohorts into the current analysis of the built environment.

A total of 2901 men and 2871 women lived in the 20 towns targeted for the environmental audit (figure 1). Of these, 1887 men and 2530 women were invited to participate in the accelerometer study. Individuals were included in the analysis if they had complete data on PA outcomes, exposures of interest and confounders and if they were not home bound, resulting in 1433 participants (795 men and 638 women ranging in age from 69 to 92 years).

Median time spent in MVPA was 30.1 min/day (lower quartile (LQ)=14.5, upper quartile (UQ)=52.7) in men and 25.5 min/day (LQ=13.4, UQ=48.1) in women, and mean time spent in LIPA was 194.3 min/day (SD 63.7) in men and 224.8 min/day (SD 63.7) in women. Median step count was 4232 (LQ=2797, UQ=6323) for men and 4148 (LQ=2799, UQ=6065) for women. Total step count was highly correlated with time spent in MVPA (Pearson correlation coefficient: 0.9). Descriptive statistics for the area-level variables are given in online supplementary material table S4.

10.1136/jech-2017-209440.supp2 Supplementary file 2

Most of the confounding factors were associated with PA in the expected directions: levels of PA declined with age and with reported long-standing illness, disability or infirmity (tables 1–3). For individual social class, the association was more mixed but in general higher levels of PA were observed for individuals in social classes I and II compared with IV and V. MVPA and step count were similar across genders, but LIPA was higher in women. No convincing evidence of interactions between environmental exposures and either gender or season of accelerometer measurement was found, and there was no evidence of effect modification by car usage for any of the outcome–exposure combinations (data not shown).

**Table 1:** Associations between the local environment and time spent in moderate to vigorous physical activity (minutes per day)

	n (%)	Geometric mean (SD) MVPA	Minimally adjusted			Confounder adjusted			Mutually adjusted		
			Relative diff.	95% CI	P value (trend)	Relative diff.	95% CI	P value (trend)	Relative diff.	95% CI	P value (trend)
Area-level exposures of interest											
Road quality score*					0.82			0.49			0.35
0 (worst quality walking environment)	415 (29.0)	24.72 (2.66)	1.00	(ref)		1.00	(ref)		1.00	(ref)	
1	530 (37.0)	23.79 (2.58)	0.95	0.84 to 1.07		0.98	0.88 to 1.09		0.98	0.88 to 1.09	
2 (best quality walking environment)	488 (34.1)	25.62 (2.55)	0.99	0.87 to 1.12		1.04	0.93 to 1.16		1.05	0.94 to 1.18	
Transport					0.94			0.88			0.73
0 (fewest bus stops)	454 (31.7)	24.16 (2.81)	1.00	(ref)		1.00	(ref)		1.00	(ref)	
1	539 (37.6)	25.39 (2.47)	1.05	0.93 to 1.18		1.05	0.94 to 1.16		1.01	0.89 to 1.14	
2 (most bus stops)	440 (30.7)	24.33 (2.51)	0.99	0.88 to 1.13		1.01	0.90 to 1.12		0.98	0.85 to 1.13	
Aesthetics†					0.76			0.97			0.95
0 (worst aesthetics)	564 (39.4)	24.68 (2.60)	1.00	(ref)		1.00	(ref)		1.00	(ref)	
1	484 (33.8)	24.43 (2.69)	1.01	0.90 to 1.14		0.98	0.89 to 1.09		0.93	0.81 to 1.06	
2 (best aesthetics)	385 (26.9)	24.96 (2.46)	1.02	0.90 to 1.15		1.00	0.90 to 1.11		0.99	0.84 to 1.17	
Shops and services					0.88			0.48			0.28
0 (fewest shops and services)	515 (35.9)	24.39 (2.74)	1.00	(ref)		1.00	(ref)		1.00	(ref)	
1	552 (38.5)	25.12 (2.50)	1.02	0.91 to 1.14		1.03	0.93 to 1.14		1.05	0.94 to 1.18	
2 (most shops and services)	366 (25.5)	24.39 (2.52)	0.99	0.87 to 1.12		1.04	0.93 to 1.16		1.08	0.94 to 1.24	
Green areas					0.99			0.94			0.95
0 (fewest green areas)	518 (36.2)	24.80 (2.45)	1.00	(ref)		1.00	(ref)		1.00	(ref)	
1	462 (32.2)	24.43 (2.69)	0.97	0.85 to 1.09		1.02	0.91 to 1.13		1.01	0.91 to 1.13	
2 (most green areas)	453 (31.6)	24.76 (2.66)	1.00	0.88 to 1.14		1.00	0.90 to 1.12		1.00	0.90 to 1.12	
Income‡					0.01			0.05			0.07
0 (least deprived)	745 (52.0)	26.24 (2.61)	1.00	(ref)		1.00	(ref)		1.00	(ref)	
1	395 (27.6)	24.59 (2.44)	0.95	0.85 to 1.07		0.96	0.87 to 1.07		0.95	0.85 to 1.07	
2 (most deprived)	293 (20.5)	21.17 (2.72)	0.84	0.74 to 0.95		0.89	0.79 to 1.00		0.87	0.75 to 1.01	
Crime‡					0.37			0.34			0.68
0 (lowest crime)	667 (46.6)	25.96 (2.61)	1.00	(ref)		1.00	(ref)		1.00	(ref)	
1	434 (30.3)	23.82 (2.56)	0.93	0.83 to 1.04		0.96	0.87 to 1.06		0.97	0.87 to 1.08	
2 (highest crime)	332 (23.2)	23.30 (2.58)	0.95	0.84 to 1.09		0.96	0.86 to 1.08		0.98	0.85 to 1.12	
Walkability§					0.75			0.94			0.83
0 (lowest walkability)	393 (27.4)	24.90 (2.71)	1.00	(ref)		1.00	(ref)		1.00	(ref)	
1	552 (38.5)	24.46 (2.46)	0.99	0.87 to 1.12		1.03	0.93 to 1.15		1.00	0.86 to 1.15	
2 (highest walkability)	488 (34.1)	24.72 (2.65)	0.98	0.86 to 1.11		1.01	0.90 to 1.13		1.02	0.85 to 1.22	
Population density¶					0.34			0.71			0.80
0 (lowest population density)	460 (32.1)	24.38 (2.66)	1.00	(ref)		1.00	(ref)		1.00	(ref)	
1	523 (36.5)	26.27 (2.53)	1.07	0.95 to 1.20		1.09	0.98 to 1.21		1.11	0.96 to 1.29	
2 (highest population density)	450 (31.4)	23.21 (2.58)	0.94	0.83 to 1.06		0.98	0.88 to 1.09		1.00	0.83 to 1.22	
Confounders											
Sex (study)					0.98						
Female (BWHHS)	638 (44.5)	23.79 (2.47)	1.00	(ref)							
Male (BRHS)	795 (55.5)	25.40 (2.68)	1.00	0.90 to 1.11							
Age (years)					<0.001						
<75	470 (32.8)	36.59 (2.28)	1.00	(ref)							
75–79	492 (34.3)	27.86 (2.34)	0.77	0.69 to 0.85							
80–84	334 (23.3)	16.75 (2.44)	0.47	0.42 to 0.53							
85+	137 (9.6)	10.59 (2.68)	0.31	0.26 to 0.36							
Adult social class					0.18						
I (professional)/II (intermediate)	631 (44.0)	26.12 (2.52)	1.00	(ref)							
IIInm (skilled non-manual)	248 (17.3)	22.24 (2.55)	0.89	0.78 to 1.02							
IIIm (skilled manual)	389 (27.2)	25.59 (2.68)	1.01	0.90 to 1.13							
IV (partially skilled manual)/V (unskilled manual)	165 (11.5)	21.25 (2.65)	0.89	0.76 to 1.04							
Long-standing illness, disability or infirmity					<0.001						
No	920 (64.2)	29.70 (2.40)	1.00	(ref)							
Yes	513 (35.8)	17.68 (2.70)	0.61	0.56 to 0.68							
Country					0.004						
England	1305 (91.1)	24.08 (2.60)	1.00	(ref)							
Scotland	128 (8.9)	31.58 (2.44)	1.32	1.09 to 1.60							

Multilevel linear regression models with random intercepts at the town and LSOA/datazone levels. Restricted to study members non-missing for all variables in the table (n=1433 study members (638 women, 795 men) across 618 LSOAs/data zones with median 2 (range 1–16) study members per LSOA/data zone and with median 78 (range 17–120) study members per town).

All models adjusted for actigraph wear time and season of physical activity data collection (season defined as spring (March to May), summer (June to August), autumn (September to November) and winter (December to February).

*Road quality score calculated from latent class analysis including 10 variables: ‘quality of pavement’; ‘lowered curbs’; ‘barriers on pavement’; ‘pavement width’; ‘pedestrian traffic’; ‘road use’; ‘road connectivity’; ‘traffic calming measures’; ‘lamp posts’ and ‘road crossings’ (full details in online supplementary material).

†Variables included in aesthetic score = ‘neighbourhood watch signs’; ‘security measures’; greenery factors’; ‘graffiti’ and ‘litter/dog foul’.

‡Income deprivation score and crime score generated from the 2010 Index of Multiple Deprivation LSOA rank (IMD: www.gov.co.uk/government/statistics/english-indices-of-deprivation-2010) or the 2009 Scottish Index of Multiple Deprivation datazone rank (SIMD: http://www.gov.scot/Topics/Statistics/SIMD) to define relative deprivation of an area for England and Scotland respectively.

§Area walkability generated from street connectivity defined as the number of road nodes/interconnections per km^2^ within an LSOA/datazone obtained from 2015 Ordinance Survey (Digimap Meridian 2 National).

¶Population density obtained from mid-year population estimates from 2010 from the Office of National Statistics (www.ons.gov.uk) and the Scottish Neighbourhood Statistics (www.sns.gov.uk). Estimates used to generate population density per km^2^ at the area level smoothed using a 5 km radius buffer.

BRHS, British Regional Heart Study; BWHHS, British Womens’ Heart and Health Study; LSOA, Lower Layer Super Output Areas.

**Table 2:** Associations between the local environment and time spent in low intensity physical activity (minutes per day)

	n (%)	Mean (SD) LIPA	Minimally adjusted			Confounder adjusted			Mutually adjusted		
			Diff.	95% CI	P value (trend)	Diff.	95% CI	P value (trend)	Diff.	95% CI	P value (trend)
Area-level exposures of interest											
Road quality score*					0.02			0.04			0.09
0 (worst quality walking environment)	415 (29.0)	212.27 (67.80)	0.00	(ref)		0.00	(ref)		0.00	(ref)	
1	530 (37.0)	206.44 (66.46)	−5.98	−13.79 to 1.83		−3.48	−10.60 to 3.65		−3.23	−10.54 to 4.08	
2 (best quality walking environment)	488 (34.1)	205.73 (62.19)	−10.50	−18.94 to –2.07		−8.10	−15.68 to 0.53		−6.98	−15.00 to 1.05	
Transport					0.27			0.21			0.94
0 (fewest bus stops)	454 (31.7)	209.21 (67.44)	0.00	(ref)		0.00	(ref)		0.00	(ref)	
1	539 (37.6)	210.69 (65.44)	2.89	−4.72 to 10.49		2.00	−4.96 to 8.95		5.00	−3.36 to 13.37	
2 (most bus stops)	440 (30.7)	203.08 (63.23)	−4.71	−12.82 to 3.40		−4.80	−12.22 to 2.61		1.14	−8.52 to 10.80	
Aesthetics†					0.25			0.02			0.19
0 (worst aesthetics)	564 (39.4)	213.11 (65.99)	0.00	(ref)		0.00	(ref)		0.00	(ref)	
1	484 (33.8)	205.87 (66.09)	−2.66	−10.25 to 4.93		−3.90	−10.79 to 3.00		−4.98	−14.14 to 4.18	
2 (best aesthetics)	385 (26.9)	202.77 (63.45)	−4.84	−13.11 to 3.42		−8.68	−16.11 to 1.25		−7.88	−19.44 to 3.68	
Shops and services					0.43			0.29			0.72
0 (fewest shops and services)	515 (35.9)	209.14 (65.38)	0.00	(ref)		0.00	(ref)		0.00	(ref)	
1	552 (38.5)	208.97 (66.28)	−0.36	−7.72 to 6.99		−0.68	−7.43 to 6.08		2.11	−5.62 to 9.83	
2 (most shops and services)	366 (25.5)	204.49 (64.35)	−3.46	−11.65 to 4.73		−4.21	−11.71 to 3.28		1.71	−7.54 to 10.96	
Green areas					0.92			0.77			0.67
0 (fewest green areas)	518 (36.2)	207.13 (65.91)	0.00	(ref)		0.00	(ref)		0.00	(ref)	
1	462 (32.2)	210.67 (68.45)	0.46	−7.82 to 8.75		3.71	−3.61 to 11.03		1.65	−5.92 to 9.23	
2 (most green areas)	453 (31.6)	205.92 (61.74)	−0.58	−10.11 to 8.96		0.96	−7.15 to 9.08		−1.86	−10.23 to 6.50	
Income‡					0.06			0.04			0.29
0 (least deprived)	745 (52.0)	212.65 (65.98)	0.00	(ref)		0.00	(ref)		0.00	(ref)	
1	395 (27.6)	206.65 (64.41)	−2.72	−10.26 to 4.82		−4.15	−11.02 to 2.73		−2.50	−10.11 to 5.10	
2 (most deprived)	293 (20.5)	197.44 (64.41)	−8.46	−16.99 to 0.08		−8.23	−16.19 to 0.28		−5.25	−15.12 to 4.63	
Crime‡					0.53			0.17			0.70
0 (lowest crime)	667 (46.6)	211.86 (66.80)	0.00	(ref)		0.00	(ref)		0.00	(ref)	
1	434 (30.3)	206.47 (65.59)	−2.79	−10.27 to 4.70		−3.48	−10.30 to 3.34		−1.21	−8.61 to 6.18	
2 (highest crime)	332 (23.2)	201.76 (62.09)	−2.40	−11.44 to 6.64		−5.24	−13.18 to 2.70		−1.81	−11.59 to 7.96	
Walkability§					0.12			0.09			0.86
0 (lowest walkability)	393 (27.4)	213.69 (67.27)	0.00	(ref)		0.00	(ref)		0.00	(ref)	
1	552 (38.5)	206.66 (64.61)	−4.99	−12.94 to 2.96		−1.80	−9.06 to 5.47		0.13	−9.59 to 9.86	
2 (highest walkability)	488 (34.1)	204.60 (64.75)	−6.73	−15.02 to 1.56		−6.40	−13.97 to 1.16		1.01	−11.30 to 13.32	
Population density¶					0.03			0.04			0.81
0 (lowest population density)	460 (32.1)	213.16 (67.47)	0.00	(ref)		0.00	0.00 to 0.00		0.00	0.00 to 0.00	
1	523 (36.5)	208.71 (64.56)	−3.44	−11.14 to 4.26		−2.24	−9.29 to 4.82		0.35	−9.82 to 10.51	
2 (highest population density)	450 (31.4)	201.54 (63.99)	−8.88	−16.99 to –0.76		−7.97	−15.41 to 0.54		−1.31	−14.33 to 11.70	
Confounders											
Sex (study)					<0.001						
Female (BWHHS)	638 (44.5)	224.80 (63.70)	0.00	(ref)							
Male (BRHS)	795 (55.5)	194.31 (63.70)	−38.28	−44.79 to –31.77							
Age (years)					<0.001						
<75	470 (32.8)	228.68 (61.97)	0.00	(ref)							
75–79	492 (34.3)	212.27 (62.41)	−13.60	−20.84 to –6.35							
80–84	334 (23.3)	188.65 (62.07)	−35.12	−43.13 to –27.12							
85+	137 (9.6)	167.71 (65.37)	−52.82	−63.74 to–41.90							
Adult social class					0.74						
I (professional)/II (intermediate)	631 (44.0)	211.00 (63.72)	0.00	(ref)							
IIInm (skilled non-manual)	248 (17.3)	212.87 (67.88)	5.45	−3.33 to 14.23							
IIIm (skilled manual)	389 (27.2)	204.80 (65.63)	−0.41	−8.12 to 7.30							
IV (partially skilled manual)/V (unskilled manual)	165 (11.5)	195.76 (66.60)	−4.84	−15.26 to 5.58							
Long-standing illness, disability or infirmity					<0.001						
No	920 (64.2)	218.46 (63.09)	0.00	(ref)							
Yes	513 (35.8)	188.93 (65.40)	−24.88	−31.20 to –18.56							
Country					0.33						
England	1305 (91.1)	207.10 (65.74)	0.00	(ref)							
Scotland	128 (8.9)	215.86 (62.18)	10.60	−10.88 to 32.08							

Multilevel linear regression models with random intercepts at the town and LSOA/data zone levels. Restricted to study members non-missing for all variables in the table (n=1433 study members (638 women, 795 men) across 618 LSOAs/data zones with median 2 (range 1–16) study members per LSOA/data zone and with median 78 (range 17–120) study members per town).

All models adjusted for actigraph wear time and season of physical activity data collection (season defined as spring (March to May), summer (June to August), autumn (September to November) and winter (December to February).

*Road quality score calculated from latent class analysis including 10 variables: ‘quality of pavement’; ‘lowered curbs’; ‘barriers on pavement’; ‘pavement width’; ‘pedestrian traffic’; ‘road use’; ‘road connectivity’; ‘traffic calming measures’; ‘lamp posts’ and ‘road crossings’ (full details in online supplementary material).

†Variables included in aesthetic score = ‘neighbourhood watch signs’; ‘security measures’; greenery factors’; ‘graffiti’ and ‘litter/dog foul’.

‡Income deprivation score and crime score generated from the 2010 Index of Multiple Deprivation LSOA rank (IMD: www.gov.co.uk/government/statistics/english-indices-of-deprivation-2010) or the 2009 Scottish Index of Multiple Deprivation datazone rank (SIMD: http://www.gov.scot/Topics/Statistics/SIMD) to define relative deprivation of an area for England and Scotland respectively.

§Area walkability generated from street connectivity defined as the number of road nodes/interconnections per km^2^ within an LSOA/datazone obtained from 2015 Ordinance Survey (Digimap Meridian 2 National).

¶Population density obtained from mid-year population estimates from 2010 from the Office of National Statistics (www.ons.gov.uk) and the Scottish Neighbourhood Statistics (www.sns.gov.uk). Estimates used to generate population density per km^2^ at the area level smoothed using a 5 km radius buffer.

BRHS, British Regional Heart Study; BWHHS, British Womens’ Heart and Health Study; LSOA, Lower Layer Super Output Areas.

**Table 3:** Associations between the local environment and total step count (steps per day)

	n (%)	Geometric mean (SD) steps	Minimally adjusted			Confounder adjusted			Mutually adjusted		
			Relative diff.	95% CI	P value (trend)	Relative diff.	95% CI	P value (trend)	Relative diff.	95% CI	P value (trend)
Area-level exposures of interest											
Road quality score*					0.66			0.65			0.45
0 (worst quality walking environment)	415 (29.0)	4026.92 (1.82)	1.00	(ref)		1.00	(ref)		1.00	(ref)	
1	530 (37.0)	3831.96 (1.90)	0.95	0.87 to 1.02		0.97	0.90 to 1.03		0.97	0.90 to 1.04	
2 (best quality walking environment)	488 (34.1)	4107.40 (1.79)	0.98	0.90 to 1.07		1.02	0.95 to 1.09		1.03	0.95 to 1.11	
Transport					0.57			0.80			0.90
0 (fewest bus stops)	454 (31.7)	3944.57 (1.87)	1.00	(ref)		1.00	(ref)		1.00	(ref)	
1	539 (37.6)	4071.27 (1.82)	1.03	0.95 to 1.11		1.03	0.97 to 1.10		1.02	0.95 to 1.11	
2 (most bus stops)	440 (30.7)	3908.18 (1.84)	0.98	0.90 to 1.06		0.99	0.92 to 1.06		1.00	0.91 to 1.09	
Aesthetics†					0.98			0.65			0.98
0 (worst aesthetics)	564 (39.4)	4011.37 (1.81)	1.00	(ref)		1.00	(ref)		1.00	(ref)	
1	484 (33.8)	3961.59 (1.90)	1.01	0.94 to 1.09		0.99	0.93 to 1.06		0.98	0.90 to 1.06	
2 (best aesthetics)	385 (26.9)	3959.01 (1.81)	1.00	0.92 to 1.08		0.98	0.92 to 1.06		1.00	0.90 to 1.11	
Shops and services					0.91			0.53			0.20
0 (fewest shops and services)	515 (35.9)	4000.38 (1.85)	1.00	(ref)		1.00	(ref)		1.00	(ref)	
1	552 (38.5)	3949.88 (1.85)	0.99	0.92 to 1.06		0.99	0.93 to 1.06		1.01	0.94 to 1.08	
2 (most shops and services)	366 (25.5)	3998.67 (1.81)	1.00	0.92 to 1.08		1.03	0.96 to 1.10		1.06	0.97 to 1.15	
Green areas					0.94			0.69			0.88
0 (fewest green areas)	518 (36.2)	3952.02 (1.83)	1.00	(ref)		1.00	(ref)		1.00	(ref)	
1	462 (32.2)	3983.97 (1.87)	0.99	0.91 to 1.07		1.03	0.96 to 1.10		1.02	0.95 to 1.09	
2 (most green areas)	453 (31.6)	4009.48 (1.82)	1.00	0.91 to 1.09		1.01	0.94 to 1.09		1.01	0.93 to 1.08	
Income‡					0.003			0.02			0.03
0 (least deprived)	745 (52.0)	4164.28 (1.83)	1.00	(ref)		1.00	(ref)		1.00	(ref)	
1	395 (27.6)	3999.60 (1.77)	0.97	0.90 to 1.05		0.98	0.92 to 1.05		0.97	0.91 to 1.05	
2 (most deprived)	293 (20.5)	3525.71 (1.93)	0.87	0.80 to 0.95		0.91	0.84 to 0.98		0.90	0.82 to 0.98	
Crime‡					0.46			0.34			0.89
0 (lowest crime)	667 (46.6)	4126.41 (1.87)	1.00	(ref)		1.00	(ref)		1.00	(ref)	
1	434 (30.3)	3918.58 (1.78)	0.97	0.90 to 1.04		0.98	0.92 to 1.05		1.00	0.93 to 1.07	
2 (highest crime)	332 (23.2)	3779.09 (1.85)	0.97	0.89 to 1.06		0.97	0.90 to 1.04		0.99	0.91 to 1.09	
Walkability§					0.40			0.59			0.87
0 (lowest walkability)	393 (27.4)	4088.54 (1.83)	1.00	(ref)		1.00	(ref)		1.00	(ref)	
1	552 (38.5)	3916.07 (1.79)	0.97	0.89 to 1.05		0.99	0.93 to 1.06		0.98	0.90 to 1.07	
2 (highest walkability)	488 (34.1)	3967.90 (1.91)	0.96	0.89 to 1.05		0.98	0.91 to 1.05		1.00	0.90 to 1.13	
Population density¶					0.11			0.25			0.49
0 (lowest population density)	460 (32.1)	4026.53 (1.83)	1.00	(ref)		1.00	(ref)		1.00	(ref)	
1	523 (36.5)	4105.02 (1.84)	1.01	0.94 to 1.09		1.02	0.96 to 1.10		1.03	0.94 to 1.14	
2 (highest population density)	450 (31.4)	3795.39 (1.85)	0.94	0.86 to 1.01		0.96	0.89 to 1.03		0.97	0.86 to 1.10	
Confounders											
Sex (study)					0.12						
Female (BWHHS)	638 (44.5)	3976.83 (1.78)	1.00	(ref)							
Male (BRHS)	795 (55.5)	3983.29 (1.89)	0.95	0.89 to 1.01							
Age (years)					<0.001						
<75	470 (32.8)	5139.05 (1.67)	1.00	(ref)							
75–79	492 (34.3)	4322.75 (1.70)	0.85	0.80 to 0.91							
80–84	334 (23.3)	3101.12 (1.78)	0.62	0.58 to 0.67							
85+	137 (9.6)	2264.11 (1.96)	0.47	0.42 to 0.52							
Adult social class					0.12						
I (professional)/II(intermediate)	631 (44.0)	4171.57 (1.80)	1.00	(ref)							
IIInm (skilled non-manual)	248 (17.3)	3720.68 (1.79)	0.93	0.85 to 1.01							
IIIm (skilled manual)	389 (27.2)	4038.95 (1.91)	1.00	0.93 to 1.07							
IV (partially skilled manual)/V (unskilled manual)	165 (11.5)	3557.33 (1.88)	0.92	0.84 to 1.02							
Long-standing illness, disability or infirmity					<0.001						
No	920 (64.2)	4507.46 (1.74)	1.00	(ref)							
Yes	513 (35.8)	3184.78 (1.90)	0.73	0.69 to 0.77							
Country					0.01						
England	1305 (91.1)	3915.01 (1.85)	1.00	(ref)							
Scotland	128 (8.9)	4712.91 (1.74)	1.21	1.05 to 1.39							

Multilevel linear regression models with random intercepts at the town and LSOA/data zone levels. Restricted to study members non-missing for all variables in the table (n=1433 study members (638 women, 795 men) across 618 LSOAs/data zones with median 2 (range 1–16) study members per LSOA/data zone and with median 78 (range 17–120) study members per town).

All models adjusted for actigraph wear time and season of physical activity data collection (season defined as spring (March to May), summer (June to August), autumn (September to November) and winter (December to February).

*Road quality score calculated from latent class analysis including 10 variables: ‘quality of pavement’; ‘lowered curbs’; ‘barriers on pavement’; ‘pavement width’; ‘pedestrian traffic’; ‘road use’; ‘road connectivity’; ‘traffic calming measures’; ‘lamp posts’ and ‘road crossings’ (full details in online supplementary material).

†Variables included in aesthetic score = ‘neighbourhood watch signs’; ‘security measures’; greenery factors’; ‘graffiti’ and ‘litter/dog foul’.

‡Income deprivation score and crime score generated from the 2010 Index of Multiple Deprivation LSOA rank (IMD: www.gov.co.uk/government/statistics/english-indices-of-deprivation-2010) or the 2009 Scottish Index of Multiple Deprivation datazone rank (SIMD: http://www.gov.scot/Topics/Statistics/SIMD) to define relative deprivation of an area for England and Scotland respectively.

§Area walkability generated from street connectivity defined as the number of road nodes/interconnections per km^2^ within an LSOA/datazone obtained from 2015 Ordinance Survey (Digimap Meridian 2 National).

¶Population density obtained from mid-year population estimates from 2010 from the Office of National Statistics (www.ons.gov.uk) and the Scottish Neighbourhood Statistics (www.sns.gov.uk). Estimates used to generate population density per km^2^ at the area level smoothed using a 5 km radius buffer.

BRHS, British Regional Heart Study; BWHHS, British Womens’ Heart and Health Study; LSOA, Lower Layer Super Output Areas.

There was no evidence that any of the physical environment domains captured by the audit tool were associated with time spent in MVPA (table 1). There was some suggestion that areas ranked higher for income deprivation were associated with lower levels of activity (14% (95% CI 5 to 26) less time spent in MVPA in the most deprived compared with the least deprived areas) (table 1).

There was no strong evidence of association between LIPA and any of the physical environmental domains (table 2). There was a suggestion that better quality walking environments (captured by the road quality score) and higher levels of area income deprivation were associated with lower levels of LIPA (10.5 (95% CI: 2.1 to 18.9) min/day lower LIPA in the highest road quality score group relative to the lowest group; 8.5 (95% CI: 0.1 to 17.0) min/day lower LIPA in the most deprived compared with the least deprived areas), though these were largely attenuated on adjustment. Area population density was inversely associated with time in LIPA (8.9 (95% CI: 0.8 to 17.0) min/day lower LIPA in areas with highest population density relative to the lowest group), although the association was no longer apparent in mutually adjusted models (table 2).

Daily step count showed no association with any of the physical environment domains considered (table 3), although it was associated with area income deprivation; relative to the least deprived group, the most deprived were estimated to have 13% (95% CI: 5 to 20) lower step count (table 3).

We found little evidence for associations between the individual components of the road quality score with either the primary or secondary outcomes (online supplementary table S5-S7). There was suggestive evidence that narrower pavements, lower amounts of pedestrian traffic and fewer traffic calming measures were associated with greater levels of LIPA (online supplementary table S6). However, these findings should be interpreted with caution due to the extent of multiple testing.

## Discussion

This is one of the largest studies worldwide to use both objectively measured PA and fine-detail neighbourhood audits within a national study of older men and women. We observed no associations between various physical environment domains and the primary outcome, time spent in MVPA, although analysis of income deprivation suggests that the social environment may be associated with PA in this age group.

This field comprises many different research approaches making comparisons challenging.^18^ A previous study reporting objective PA in older adults, but with areas selected for either high or low walkability, also found no association with MVPA but reported an inverse association with LIPA and interaction with neighbourhood SES.^29^ In our analysis, there was a suggestion of an inverse association between road quality and LIPA (attenuated in fully adjusted models). We examined associations between environmental features and PA within the environments where cohort participants lived rather than selecting areas based on their environmental attributes. As a result, within-town variances in exposures were relatively narrow and may have affected the ability to detect associations. Another large multicountry study specifically selected neighbourhoods to maximise variation in walkability and SES and reported significant associations between adult PA and a number of objective features of the built environment.^30^

A previous analysis within these same two study cohorts found perceptions of ‘having somewhere nice to walk’ were associated with MVPA in men and ‘feeling safe when walking alone after dark’ were associated with MVPA in men and women.^7^ These contrasting results may suggest the relative importance of environmental perception on PA.

The most recent systematic review of built environment and PA in older adults reviewed 100 articles from six continents and found strong evidence of positive associations between walkability, access to destinations and services, personal safety from crime and PA.^18^ However, for 9 out of 18 environmental exposures, associations with total PA differed by environmental measurement type. For five, perception of crime-related safety, access to recreational facilities, open space, aesthetically pleasing scenery and destination diversity, positive associations with total PA were found with perceived but not objectively assessed environmental measures. Objectively assessed availability of shops, public transport, presence of walk-friendly infrastructure and absence of physical environmental barriers were all positively associated with total PA, whereas associations were non-significant using perceived measures.^18^ This does not necessarily mean that one type of measurement is better, but in some attributes perceived environmental measures may be more closely associated with PA than objective equivalents. Understanding these differences could inform better policy and intervention development for older age groups.

In common with other studies, we found greater income deprivation, measured by area-level statistics, was associated with less time spent in MVPA (somewhat attenuated on adjustment) and a lower step count. Area-level SES was the strongest, most common predictor of a variety of health outcomes reviewed by Yen *et al.* 9 Other studies have reported a positive association between neighbourhood income and PA in older adults,^31^ although this finding is not universal.^10^ It is important to consider how these ‘compositional factors’ (such as the collective social functioning of an area) exert an influence on behaviour and to consider the reciprocal relationships between people and place.^32 33^ A qualitative Global Positioning System (GPS) substudy conducted within this study showed that older individuals are often accessing destinations, services and social resources at various distances from their home depending on their social activity spaces.^34^ Local destinations may provide a location for social engagement encouraging physical activity.

Socioecological frameworks allow for the mechanisms through which the built environment influences PA to be understood.^11^ Barnett *et al* suggested that nearly all built environmental correlates of older adults’ total PA were also identified as being environmental correlates of either active transport and/or leisure-time PA,^18^ thus potentially explaining the behavioural pathways through which the neighbourhood impacts on total PA.

The strengths of our study include the large national sample of older men and women, objective PA measurements and a standardised audit tool designed to collect detailed objective neighbourhood environment data relevant for older people. Many previous studies have relied either on routine data or generic data capture tools.^19^ Our analysis used multilevel modelling to account for exposures operating at different scales. The resulting CIs around our effect estimates are relatively narrow giving confidence to the null results reported.

### Limitations

Although situated within two large national cohort studies, this study uses cross-sectional analysis that has clear limitations when assessing something as dynamic as the local environment. The PA data were collected prior to the environmental audits although there is limited evidence that environmental changes would have occurred over such a short time frame.^35^ Also, we were unable to look at PA disaggregated into domains (eg, walking for transport) and this lack of specificity may have weakened any potential relationship between PA and environment. No adjustment was possible in the analysis to take account of neighbourhood self-selection. While the environment score was highly detailed, it was novel and had not previously been validated. Finally, we used lower-level administrative boundaries as the unit of analysis that are arbitrary with respect to exposures of interest. Analysing exposure at a fixed spatial area may underestimate the total effect of contextual factors,^33^ and our null results may reflect this spatial scale. A qualitative GPS substudy showed older individuals are often using services and social resources at various distances from their home.^34^ Other studies suggest that older individuals access areas wider than those captured by standard buffer sizes.^36 37^

As PA correlates are not consistent across different environmental measurement types,^18^ future research should consider these differences in findings and identify the mechanisms underlying them. Future studies should also strive to undertake higher quality research by implementing longitudinal research designs and adjusting for residential self-selection.

## Conclusions

Using objective assessments of PA and detailed environmental audit, we found limited evidence for important associations between the local neighbourhood physical environment and PA in older people in the UK. These data suggest that older individuals might be less affected by their physical environment and more by social environmental factors, reflecting both the functional diversity of this age group and their reasons for being active. There is increasing recognition of the importance of the urban environment for health of older people, with initiatives including WHO Age Friendly Cities.^38^ As individuals age a combination of decline in physical and cognitive function, and potentially reduced social networks, could lead to greater dependence on their residential neighbourhood.^9^ A supportive neighbourhood environment may be important, but not sufficient, for increasing PA in older people.

**What is already known on this subject**

Physical activity (PA) is known to be influenced by environmental factors. The most recent systematic review in older people found positive associations between neighbourhood walkability, access to destinations and services, personal safety from crime and PA, although associations varied by type of environmental measurement.

**What this study adds**

This is one of the largest studies worldwide to use both objectively measured physical activity and fine-detail neighbourhood environmental audits developed for older people within two national cohorts of older men and women. It found that older adults were less influenced by objectively measured physical environmental factors, and more by the social environment. This is likely to reflect the varying nature of activity spaces, with social engagement encouraging physical activity.
