# Supplementary material for: Investigating associations between the built environment and physical activity among older people in 20 UK towns
Source: J Epidemiol Community Health. 2017 Nov 24;72(2):121–31. doi: 10.1136/jech-2017-209440 (PMC5800350; doi:10.1136/jech-2017-209440)
Supplement: Supplementary file 1 [file jech-2017-209440supp001.pdf]

SUPPLEMENTARY MATERIAL

Hawkesworth et al

Road and path quality latent class analysis

MATERIALS AND METHODS

Measures

The following 10 road quality variables, collected as part of the foot-based environmental audit, were included in the latent class analysis (LCA): 'quality of pavement', 'lowered curbs', 'barriers on pavement', 'pavement width', 'pedestrian traffic', 'road use', 'road connectivity', 'traffic calming measures', 'lamp posts' and 'road crossings'. The first 9 of these variables were originally collected as 4- or 5-level categorical variables and were collapsed to 3-level categorical variables to avoid small numbers (or to binary variables if small numbers were still unavoidable). 'Road crossings' was originally collected as a count of the number of crossings. As the majority of segments had no crossings this was analysed as 'none' vs. 'one or more'. Segments with data on at least 7 of the 10 road quality variables were included in the LCAs.

Statistical analysis

LCA is a multivariate regression approach that describes the relationship between an unobserved categorical latent variable, each level of which is a 'latent class', and a set of observed dependent variables ('latent class indicators'), in this case the ten road quality variables. The resultant latent classes identify subgroups that have similar patterns of the latent class indicators (natural clusterings of features of the built environment in this instance). The objective is usually to identify the latent class indicators that best distinguish between classes and to categorise the units of analysis into their most likely classes given their observed responses.[1]

LCA was conducted on the 10 road quality variables identified above. The expectation-maximization (EM) algorithm[2] was used to allow the inclusion of segments with incomplete road quality data. The EM algorithm relies on the assumption that data are missing at random.[3] To ensure a true (rather than local) maximum likelihood solution had been reached we used 100, 1,000 or 10,000 random start values (as necessary) for the estimation of each model.

We used several different tools to decide how many classes were appropriate as no single approach is agreed upon as optimal.[1] The standard likelihood ratio test (LRT) is invalid in the LCA setting, so we used the Lo-Mendell-Rubin adjusted LRT.[4] We also used three information criteria: Akaike's Information Criterion (AIC),[5] Schwarz's Bayesian Information Criterion (BIC),[6] and sample size-adjusted BIC (aBIC).[7] Models that best combine goodness-of-fit and parsimony are indicated by lower values of the information criteria. In addition, the relative class sizes (calculated based on most likely class membership), meaningful interpretation of the latent classes and entropy were considered. Entropy is a summary statistic, based on the posterior class membership probabilities, which evaluates the clarity of the separation of the latent classes, with values ranging from 0 (unclear classification) to 1 (clear classification).[8]

The LCAs were conducted on all segments audited with the foot-based environmental tool (irrespective of whether they would be included in the main analysis) and were conducted using Mplus 6.[9]

RESULTS

A total of 16,520 segments were eligible for inclusion in the LCA. Of these, 16,010 (96.9%) segments with data on at least 7 of the 10 road quality variables (14,909 with complete data on all 10 variables) were included in the LCA.

The distributions of the road quality variables are given in Table S1.

Model fit statistics for the LCA are given in Table S2. Although the Lo-Mendell-Rubin LRT suggested continuing to add more classes, the information criteria showed only moderate decreases beyond 3 classes. The 3-class model also provided more easily interpretable classes and with higher entropy that models with more than 3 classes. On the balance of evidence, the 3-class model was chosen as most appropriate.

The class-specific latent class indicator probabilities for the 3-class model are given in Table S3. Class 1 (9.9%) was more likely to have no pavements, have no pedestrian traffic, be a cul-de-sac with pedestrian throughway/pedestrianised, have no/sporadically/damaged lamp posts, and have no street crossings; class 2 (57.0%) was more likely to have poor/fair quality pavement, have no/few/poor lowered curbs, have medium width pavement, have no pedestrian traffic, have no traffic calming measures, have regular lamp posts on one side, and have no road crossings; and class 3 (33.1%) was more likely to have good/excellent quality pavement, have most/all lowered curbs, have no barriers on the pavement, have wide pavement, have more pedestrian traffic, be a through road, have regular traffic calming measures, have regular lamp posts on both sides, and have one or more road crossings. There was therefore a clear ordering to the classes with class 1 representing a "poor quality walking environment", class 2 a "medium quality walking environment" and class 3 a "good quality walking environment".

Road quality latent class was available for a median of 14 (range 1-97) segments per LSOA/datazone. To obtain an area-level measure of the road quality a value was assigned to each latent class (class 1 = 0, class 2 = 1, class 3 = 2) and the mean calculated within each LSOA/datazone to provide the “road quality score”, which was then split into thirds of the observed distribution.

REFERENCES

1. Nylund KL, Asparouhov T, Muthen BO. Deciding on the Number of Classes in Latent Class Analysis and Growth Mixture Modeling: A Monte Carlo Simulation Study. *Struct Equ Modeling*. 2007; 14(4): 535-569.

2. Dempster AP, Laird NM, Rubin DB. Maximum likelihood from incomplete data via the EM algorithm. *J R Stat Soc Series B Stat Methodol*. 1977; 39(1): 1-38.

3. Little RJA, Rubin DB. Statistical Analysis With Missing Data. New York: Wiley; 2002.

4. Lo Y, Mendell NR, Rubin DB. Testing the number of components in a normal mixture. *Biometrika*. 2001; 88(3): 767-778.

5. Akaike H. Factor analysis and AIC. *Psychometrika*. 1987; 52(3): 317-332.

6. Schwartz G. Estimating the dimension of a model. *Ann Stat*. 1978; 6(2): 461-464.

7. Sclove L. Application of model-selection criteria to some problems in multivariate analysis. *Psychometrika*. 1987; 52(3): 333-343.

8. Muthén LK, Muthén BO. Mplus User's Guide. Seventh Edition. Los Angeles, CA: Muthén & Muthén; 1998-2012.

9. Muthén & Muthén. Mplus statistical software, release 6. Los Angeles, CA: Muthén & Muthén; 2010.

TABLES

Table S1. Foot-based environmental audit road and path quality data (n = 16,010).

	n	%
Quality of pavement		
Poor/fair	6928	43.5
Good/excellent	7448	46.7
N/A	1564	9.8
Total	15,940	
Lowered curbs		
None/few/poor	6060	38.7
Most/all	7985	51.0
N/A	1599	10.2
Total	15,644	
Barriers on pavement		
None	10,377	65.5
Occasional/often	3908	24.7
N/A	1555	9.8
Total	15,840	
Pavement width		
None/narrow/1 person/variable	3509	21.9
2 people	10,159	63.5
> 2 people	2323	14.5
Total	15,991	
Pedestrian traffic		
No people	4213	26.5
Few	10,628	66.9
Many/crowded	1044	6.6
Total	15,885	
Road use		
One-way	506	3.2
Two-way	15,280	96.8
Total	15,786	
Road connectivity		
Through road	12,320	77.1
Cul de sac with pedestrian throughway/pedestrianised	3658	22.9
Total	15,978	
Traffic calming measures		
Absent	12,758	81.1
At one or two points	1813	11.5
Regularly	1166	7.4
Total	15,737	
Lamp posts		

None/sporadically/damaged	2333	14.6
Regularly on one side	3881	24.4
Regularly on both sides	9713	61.0
Total	15,927	
Road crossings		
None	13,596	84.9
One or more	2410	15.1
Total	16,006	

Table S2. Foot-based environmental audit road quality latent class analysis model fit statistics. Full information maximum likelihood (n = 16,010).

	1 class	2 classes	3 classes	4 classes	5 classes	6 classes
No. parameters	17	35	53	71	89	107
Loglikelihood	-112,430	-98,760	-96,598	-96,069	-95,705	-95,479
Information criterion						
AIC	224,894	197,589	193,303	192,280	191,589	191,172
BIC	225,025	197,858	193,708	192,825	192,272	191,994
aBIC	224,971	197,747	193,541	192,600	191,989	191,654
Entropy	-	0.997	0.727	0.643	0.642	0.641
Minimum class proportion	-	0.099	0.099	0.098	0.075	0.080
Lo-Mendell-Rubin LRT p-value	-	<0.001	<0.001	<0.001	<0.001	<0.001

AIC, Akaike's Information Criterion; BIC, Bayesian Information Criterion; aBIC, sample size adjusted Bayesian Information Criterion, LRT, likelihood ratio test.

Table S3. Foot-based environmental audit class-specific latent class indicator probabilities (3 classes).

	Observed proportion	Class 1 (9.9%)	Class 2 (57.0%)	Class 3 (33.1%)
Quality of pavement				
Poor/fair	0.435	0.008	0.570	0.345
Good/excellent	0.467	0.014	0.428	0.655
N/A	0.098	0.978	0.002	0.000
Lowered curbs				
None/few/poor	0.387	0.012	0.584	0.194
Most/all	0.510	0.029	0.408	0.804
N/A	0.102	0.959	0.008	0.002
Barriers on pavement				
None	0.655	0.012	0.706	0.758
Occasional/often	0.247	0.005	0.294	0.242
N/A	0.098	0.983	0.000	0.000
Pavement width				
None/narrow/1 person/variable	0.219	0.987	0.164	0.092
2 people	0.635	0.004	0.758	0.621
> 2 people	0.145	0.010	0.078	0.287
Pedestrian traffic				
No people	0.265	0.557	0.328	0.086
Few	0.669	0.409	0.658	0.759
Many/crowded	0.066	0.034	0.014	0.155
Road use				
One-way	0.038	0.038	0.020	0.049
Two-way	0.962	0.962	0.980	0.951
Road connectivity				
Through road	0.771	0.632	0.674	0.960
Cul de sac with pedestrian throughway/pedestrianised	0.229	0.368	0.326	0.040
Traffic calming measures				
Absent	0.811	0.856	0.897	0.666
At one or two points	0.115	0.091	0.075	0.184
Regularly	0.074	0.053	0.028	0.150
Lamp posts				
None/sporadically/damaged	0.146	0.686	0.125	0.030
Regularly on one side	0.244	0.185	0.317	0.147
Regularly on both sides	0.610	0.130	0.558	0.824
Road crossings				
None	0.849	0.970	0.976	0.620
One or more	0.151	0.030	0.024	0.380
