# Supplementary material for: Investigating associations between the built environment and physical activity among older people in 20 UK towns
Source: J Epidemiol Community Health. 2017 Nov 24;72(2):121–31. doi: 10.1136/jech-2017-209440 (PMC5800350; doi:10.1136/jech-2017-209440)
Supplement: Supplementary file 2 [file jech-2017-209440supp002.pdf]

Table S4. Area-level descriptive statistics for the 618 LSOAs/datazones containing the 1433 study members (638 women, 795 men) contributing to the analyses.

Area-level exposures of interest	Variable description	Number of LSOAs	Per LSOA/datazone[a] Minimum	Lower quartile	Median	Upper quartile	Maximum
Road quality score[b]	Calculated from latent class analysis	618	0.25	1.13	1.25	1.42	2.00
Transport	Number of bus stops per km^2	618	0.0	6.9	12.6	22.3	75.2
Aesthetics[c]	Aesthetic score based on presence of 5 factors per km^2	618	0.7	137	254	391	1474
Shops and services	Number of shops and services per km^2	618	0	5	16	37	1441
Green areas	Proportion of segments with green areas	618	0.00	0.17	0.37	0.69	1.00
Income[d]							
England	Index of Multiple Deprivation 2010 income score	544	0.01	0.05	0.09	0.18	0.53
Scotland	Scottish Index of Multiple Deprivation 2009 income score	74	2	6	13	21	47
Crime[d]							
England	Index of Multiple Deprivation 2010 crime and disorder score	544	-2.5	-0.7	-0.1	0.5	2.9
Scotland	Scottish Index of Multiple Deprivation 2009 crime score	74	67	213	352	589	4760
Walkability[e]	Number of street intersections per km^2	618	3	52	104	166	571
Population density[f]	Population density per km^2	618	27	1683	3243	4750	15,082

[a]Lower-layer super output areas are the geographic unit of analysis used in England (www.ons.gov.uk) and datazones are the geographic unit of analysis used in Scotland (www.sns.gov.uk)

[b]Road quality score calculated from latent class analysis including 10 variables: 'quality of pavement'; 'lowered curbs'; 'barriers on pavement'; 'pavement width'; 'pedestrian traffic'; 'road use'; 'road connectivity'; 'traffic calming measures'; 'lamp posts' and 'road crossings' (full details in Supplementary Material)

[c]Variables included in aesthetic score = 'neighbourhood watch signs'; 'security measures'; greenery factors'; 'graffiti' and 'litter/dog foul etc'

[d]Income deprivation score and crime score generated from the 2010 Index of Multiple Deprivation LSOA rank (IMD: www.gov.co.uk/government/statistics/english-indices-of-deprivation-2010) or the 2009 Scottish Index of Multiple Deprivation datazone rank (SIMD: http://www.gov.scot/Topics/Statistics/SIMD) to define relative deprivation of an area for England and Scotland respectively

[e]Area walkability generated from street connectivity defined as the number of road nodes/interconnections per km^2 within an LSOA/datazone obtained from 2015 Ordinance Survey (Digimap Meridian 2 National)

[f]Population densitiy obtained from mid-year population estimates from 2010 from the Office of National Statistics (www.ons.gov.uk) and the Scottish Neighbourhood Statistics (www.sns.gov.uk). Estimates used to generate population density per km^2 at the area level smoothed using a 5km radius buffer.

Table S5. Associations between individual features of the road quality score and time spend in moderate-vigorous physical activity (minutes per day).

	n (%)	Geometric mean (SD) MVPA	Minimally adjusted Relative diff.	95% CI	p-value (trend)	Confounder adjusted Relative diff.	95% CI	p-value (trend)
**Area-level exposures of interest**								
Pavement quality					0.91			0.38
0 (worst quality pavements)	452 (31.5)	24.73 (2.65)	1.00	(ref)		1.00	(ref)	
1	503 (35.1)	24.18 (2.55)	0.97	0.86, 1.10		1.01	0.91, 1.13	
2 (best quality pavements)	478 (33.4)	25.13 (2.59)	1.01	0.89, 1.14		1.05	0.94, 1.17	
Lowered curbs					0.41			0.71
0 (fewest lowered curbs)	511 (35.7)	25.24 (2.54)	1.00	(ref)		1.00	(ref)	
1	442 (30.8)	25.30 (2.76)	1.00	0.88, 1.12		1.02	0.92, 1.14	
2 (most lowered curbs)	480 (33.5)	23.52 (2.50)	0.95	0.83, 1.07		1.02	0.92, 1.13	
Barriers on pavement					0.51			0.76
0 (most barriers)	434 (30.3)	26.96 (2.58)	1.00	(ref)		1.00	(ref)	
1	469 (32.7)	22.89 (2.65)	0.87	0.77, 0.99		0.89	0.80, 0.99	
2 (fewest barriers)	530 (37.0)	24.51 (2.54)	0.95	0.84, 1.08		1.01	0.90, 1.13	
Pavement width					0.64			0.41
0 (narrowest pavements)	493 (34.4)	25.33 (2.67)	1.00	(ref)		1.00	(ref)	
1	504 (35.2)	25.11 (2.46)	1.01	0.89, 1.14		1.00	0.90, 1.11	
2 (widest pavements)	436 (30.4)	23.45 (2.65)	0.97	0.85, 1.10		0.95	0.85, 1.07	
Pedestrian traffic					0.87			0.15
0 (fewest people)	486 (33.9)	24.60 (2.62)	1.00	(ref)		1.00	(ref)	
1	516 (36.0)	24.95 (2.56)	1.04	0.93, 1.18		1.09	0.98, 1.20	
2 (most people)	431 (30.1)	24.41 (2.60)	1.01	0.89, 1.15		1.08	0.97, 1.21	
Road use					0.23			0.16
0 (fewest lanes)	395 (27.6)	24.94 (2.66)	1.00	(ref)		1.00	(ref)	
1	481 (33.6)	24.85 (2.52)	1.00	0.88, 1.13		0.99	0.89, 1.11	
2 (most lanes)	557 (38.9)	24.33 (2.61)	0.93	0.82, 1.05		0.93	0.83, 1.03	
Road connectivity					0.75			0.45

0 (most through roads)	494 (34.5)	25.33 (2.53)	1.00	(ref)		1.00	(ref)	
1	501 (35.0)	23.85 (2.77)	0.95	0.84, 1.07		0.92	0.83, 1.02	
2 (fewest through roads)	438 (30.6)	24.88 (2.46)	1.02	0.90, 1.16		0.96	0.86, 1.07	
Traffic calming measures					0.82			0.96
0 (fewest traffic calming measures)	533 (37.2)	24.71 (2.50)	1.00	(ref)		1.00	(ref)	
1	524 (36.6)	24.12 (2.61)	0.96	0.86, 1.08		1.01	0.92, 1.12	
2 (most traffic calming measures)	376 (26.2)	25.40 (2.71)	0.99	0.87, 1.13		1.00	0.89, 1.12	
Lamp posts					0.17			0.21
0 (fewest lamp posts)	474 (33.1)	25.44 (2.63)	1.00	(ref)		1.00	(ref)	
1	526 (36.7)	25.49 (2.46)	1.00	0.89, 1.13		0.98	0.88, 1.09	
2 (most lamp posts)	433 (30.2)	22.93 (2.70)	0.91	0.80, 1.04		0.93	0.83, 1.04	
Crossings					0.81			0.19
0 (fewest crossings)	484 (33.8)	24.09 (2.64)	1.00	(ref)		1.00	(ref)	
1	488 (34.1)	25.07 (2.60)	1.02	0.91, 1.15		1.03	0.93, 1.15	
2 (most crossings)	461 (32.2)	24.86 (2.53)	1.01	0.90, 1.15		1.07	0.97, 1.19	

Multilevel linear regression models with random intercepts at the town and LSOA/data zone levels. Restricted to study members non-missing for all variables in the table (N = 1433 study members (638 women, 795 men) across 618 LSOAs/data zones with median 2 (range 1-16) study members per LSOA/data zone and with median 78 (range 17-120) study members per town).

All models adjusted for actigraph wear time and season of physical activity data collection (season defined as spring (March – May), summer (June – August), autumn (September – November) and winter (December to February).

Confounders = sex, age, adult social class, long-standing illness, disability or infirmity, and country of residence

Table S6. Associations between individual features of the road quality score and time spend in low intensity physical activity (minutes per day).

	n (%)	Mean (SD) LIPA	Minimally adjusted			Confounder adjusted		
			Diff.	95% CI	p-value (trend)	Diff.	95% CI	p-value (trend)
**Area-level exposures of interest**								
Pavement quality					0.06			0.12
0 (worst quality pavements)	452 (31.5)	212.47 (68.63)	0.00	0.00, 0.00		0.00	(ref)	
1	503 (35.1)	207.16 (65.70)	-6.06	-13.94, 1.82		-3.24	-10.37, 3.90	
2 (best quality pavements)	478 (33.4)	204.31 (61.90)	-7.90	-16.09, 0.30		-5.83	-13.18, 1.53	
Lowered curbs					0.07			0.43
0 (fewest lowered curbs)	511 (35.7)	211.42 (64.95)	0.00	(ref)		0.00	(ref)	
1	442 (30.8)	211.13 (70.05)	-0.25	-8.01, 7.50		1.07	-6.02, 8.16	
2 (most lowered curbs)	480 (33.5)	201.13 (61.07)	-8.52	-17.18, 0.15		-3.34	-10.91, 4.23	
Barriers on pavement					0.41			0.54
0 (most barriers)	434 (30.3)	217.25 (63.82)	0.00	(ref)		0.00	(ref)	
1	469 (32.7)	204.30 (65.12)	-6.75	-15.19, 1.70		-5.64	-13.16, 1.88	
2 (fewest barriers)	530 (37.0)	203.39 (66.39)	-4.27	-13.16, 4.63		-2.91	-10.70, 4.88	
Pavement width					0.02			0.02
0 (narrowest pavements)	493 (34.4)	215.33 (68.94)	0.00	(ref)		0.00	(ref)	
1	504 (35.2)	207.25 (62.20)	-6.33	-14.36, 1.69		-4.58	-11.76, 2.61	
2 (widest pavements)	436 (30.4)	200.20 (64.28)	-10.58	-19.38, -1.77		-9.60	-17.43, -1.76	
Pedestrian traffic					0.05			0.05
0 (fewest people)	486 (33.9)	214.52 (65.04)	0.00	(ref)		0.00	(ref)	
1	516 (36.0)	207.19 (66.06)	-1.15	-9.11, 6.81		-1.35	-8.47, 5.78	
2 (most people)	431 (30.1)	201.24 (64.61)	-8.03	-16.30, 0.24		-7.30	-14.71, 0.11	
Road use					0.68			0.75
0 (fewest lanes)	395 (27.6)	205.92 (67.17)	0.00	(ref)		0.00	(ref)	
1	481 (33.6)	204.19 (64.30)	-1.30	-9.43, 6.82		-1.19	-8.58, 6.20	
2 (most lanes)	557 (38.9)	212.47 (65.06)	1.56	-6.65, 9.78		1.08	-6.32, 8.48	
Road connectivity					0.07			0.24
0 (most through roads)	494 (34.5)	205.64 (65.30)	0.00	(ref)		0.00	(ref)	

1	501 (35.0)	210.49 (68.85)	6.87	-0.91, 14.65		3.66	-3.38, 10.70	
2 (fewest through roads)	438 (30.6)	207.44 (61.57)	7.76	-0.63, 16.14		4.40	-3.08, 11.89	
Traffic calming measures					0.004			0.02
0 (fewest traffic calming measures)	533 (37.2)	210.16 (65.59)	0.00	(ref)		0.00	(ref)	
1	524 (36.6)	209.29 (65.92)	-4.92	-12.29, 2.45		-1.28	-8.03, 5.46	
2 (most traffic calming measures)	376 (26.2)	202.70 (64.49)	-12.50	-20.96, -4.03		-9.89	-17.64, -2.14	
Lamp posts					0.13			0.20
0 (fewest lamp posts)	474 (33.1)	212.88 (64.59)	0.00	(ref)		0.00	(ref)	
1	526 (36.7)	207.22 (65.34)	-3.68	-11.38, 4.02		-4.43	-11.46, 2.60	
2 (most lamp posts)	433 (30.2)	203.23 (66.29)	-6.51	-14.86, 1.84		-4.82	-12.39, 2.75	
Crossings					0.67			0.69
0 (fewest crossings)	484 (33.8)	209.00 (65.00)	0.00	(ref)		0.00	(ref)	
1	488 (34.1)	207.73 (66.66)	-1.34	-9.10, 6.41		-0.11	-7.16, 6.95	
2 (most crossings)	461 (32.2)	206.88 (64.76)	-1.72	-9.63, 6.20		-1.50	-8.71, 5.70	

Multilevel linear regression models with random intercepts at the town and LSOA/data zone levels. Restricted to study members non-missing for all variables in the table (N = 1433 study members (638 women, 795 men) across 618 LSOAs/data zones with median 2 (range 1-16) study members per LSOA/data zone and with median 78 (range 17-120) study members per town).

All models adjusted for actigraph wear time and season of physical activity data collection (season defined as spring (March – May), summer (June – August), autumn (September – November) and winter (December to February).

Confounders = sex, age, adult social class, long-standing illness, disability or infirmity, and country of residence

Table S7. Associations between individual features of the road quality score and total step count (steps per day).

	n (%)	Geometric mean (SD) steps	Minimally adjusted Relative diff.	95% CI	p-value (trend)	Confounder adjusted Relative diff.	95% CI	p-value (trend)
**Area-level exposures of interest**								
Pavement quality					0.32			0.85
0 (worst quality pavements)	452 (31.5)	4031.10 (1.85)	1.00	1.00, 1.00		1.00	1.00, 1.00	
1	503 (35.1)	3922.91 (1.82)	0.98	0.90, 1.06		1.00	0.93, 1.07	
2 (best quality pavements)	478 (33.4)	3993.76 (1.85)	0.99	0.91, 1.07		1.01	0.95, 1.08	
Lowered curbs					0.32			0.85
0 (fewest lowered curbs)	511 (35.7)	4045.99 (1.78)	1.00	1.00, 1.00		1.00	1.00, 1.00	
1	442 (30.8)	4084.13 (1.95)	1.00	0.93, 1.09		1.02	0.96, 1.09	
2 (most lowered curbs)	480 (33.5)	3820.20 (1.79)	0.96	0.88, 1.04		1.01	0.94, 1.08	
Barriers on pavement					0.23			0.59
0 (most barriers)	434 (30.3)	4300.15 (1.78)	1.00	1.00, 1.00		1.00	1.00, 1.00	
1	469 (32.7)	3835.18 (1.84)	0.92	0.85, 1.00		0.93	0.87, 1.00	
2 (fewest barriers)	530 (37.0)	3861.31 (1.87)	0.95	0.87, 1.03		0.98	0.91, 1.05	
Pavement width					0.29			0.24
0 (narrowest pavements)	493 (34.4)	4102.96 (1.84)	1.00	(ref)		1.00	(ref)	
1	504 (35.2)	3981.11 (1.80)	0.98	0.91, 1.07		0.98	0.92, 1.05	
2 (widest pavements)	436 (30.4)	3845.46 (1.88)	0.95	0.88, 1.04		0.96	0.89, 1.03	
Pedestrian traffic					0.85			0.32
0 (fewest people)	486 (33.9)	4046.86 (1.79)	1.00	(ref)		1.00	(ref)	
1	516 (36.0)	3958.92 (1.85)	1.00	0.93, 1.08		1.03	0.96, 1.10	
2 (most people)	431 (30.1)	3932.20 (1.88)	0.99	0.91, 1.08		1.04	0.97, 1.11	
Road use					0.64			0.57
0 (fewest lanes)	395 (27.6)	3952.63 (1.90)	1.00	(ref)		1.00	(ref)	
1	481 (33.6)	3948.61 (1.84)	1.01	0.93, 1.09		1.00	0.93, 1.07	
2 (most lanes)	557 (38.9)	4028.05 (1.80)	0.98	0.91, 1.07		0.98	0.91, 1.05	

Road connectivity					0.75			0.38
0 (most through roads)	494 (34.5)	4028.02 (1.84)	1.00	(ref)		1.00	(ref)	
1	501 (35.0)	3979.96 (1.91)	1.01	0.93, 1.09		0.98	0.92, 1.05	
2 (fewest through roads)	438 (30.6)	3927.91 (1.77)	1.01	0.93, 1.10		0.97	0.90, 1.04	
Traffic calming measures					0.52			0.79
0 (fewest traffic calming measures)	533 (37.2)	3973.08 (1.82)	1.00	(ref)		1.00	(ref)	
1	524 (36.6)	3973.14 (1.88)	0.99	0.92, 1.07		1.03	0.96, 1.09	
2 (most traffic calming measures)	376 (26.2)	4001.02 (1.83)	0.97	0.89, 1.06		0.98	0.91, 1.06	
Lamp posts					0.19			0.28
0 (fewest lamp posts)	474 (33.1)	4061.94 (1.82)	1.00	(ref)		1.00	(ref)	
1	526 (36.7)	4055.23 (1.82)	1.00	0.93, 1.08		0.99	0.93, 1.06	
2 (most lamp posts)	433 (30.2)	3805.96 (1.89)	0.94	0.87, 1.03		0.96	0.90, 1.03	
Crossings					0.78			0.26
0 (fewest crossings)	484 (33.8)	3959.23 (1.84)	1.00	(ref)		1.00	(ref)	
1	488 (34.1)	3987.00 (1.83)	1.00	0.92, 1.08		1.00	0.94, 1.07	
2 (most crossings)	461 (32.2)	3995.77 (1.86)	1.01	0.93, 1.09		1.04	0.97, 1.11	

Multilevel linear regression models with random intercepts at the town and LSOA/data zone levels. Restricted to study members non-missing for all variables in the table (N = 1433 study members (638 women, 795 men) across 618 LSOAs/data zones with median 2 (range 1-16) study members per LSOA/data zone and with median 78 (range 17-120) study members per town).

All models adjusted for actigraph wear time and season of physical activity data collection (season defined as spring (March – May), summer (June – August), autumn (September – November) and winter (December to February).

Confounders = sex, age, adult social class, long-standing illness, disability or infirmity, and country of residence
